# Circular stapling anastomosis with indocyanine green fluorescence imaging for cervical esophagogastric anastomosis after thoracoscopic esophagectomy: a propensity score-matched analysis

**DOI:** 10.1186/s12893-022-01602-2

**Published:** 2022-04-29

**Authors:** Yuji Shishido, Tomoyuki Matsunaga, Masahiro Makinoya, Wataru Miyauchi, Shota Shimizu, Kozo Miyatani, Chihiro Uejima, Masaki Morimoto, Yuki Murakami, Takehiko Hanaki, Kyoichi Kihara, Manabu Yamamoto, Naruo Tokuyasu, Shuichi Takano, Teruhisa Sakamoto, Hiroaki Saito, Toshimichi Hasegawa, Yoshiyuki Fujiwara

**Affiliations:** 1 Division of Gastrointestinal and Pediatric Surgery, Department of Surgery, School of Medicine, Tottori University Faculty of Medicine, 36-1 Nishi-cho, 683-8504 Yonago, Japan; 2 Department of Surgery, Japanese Red Cross Tottori Hospital, 117 Shotoku-cho, 680- 8517 Tottori, Japan

**Keywords:** Esophageal cancer, Thoracoscopic esophagectomy, Cervical esophagogastric anastomosis, Indocyanine green fluorescence imaging, Circular stapling anastomosis, Anastomotic leakage, Anastomotic stenosis, Propensity score matching

## Abstract

**Background:**

Thoracoscopic esophagectomy has been extensively used worldwide as a curative surgery for patients with esophageal cancer; however, complications such as anastomotic leakage and stenosis remain a major concern. Therefore, the objective of this study was to evaluate the efficacy of circular stapling anastomosis with indocyanine green (ICG) fluorescence imaging, which was standardized for cervical esophagogastric anastomosis after thoracoscopic esophagectomy.

**Methods:**

Altogether, 121 patients with esophageal cancer who underwent thoracoscopic esophagectomy with radical lymph node dissection and cervical esophagogastric anastomosis from November 2009 to December 2020 at Tottori University Hospital were enrolled in this study. Patients who underwent surgery before the anastomotic method was standardized were included in the classical group (n = 82) and patients who underwent surgery after the anastomotic method was standardized were included in the ICG circular group (n = 39). The short-term postoperative outcomes, including anastomotic complications, were compared between the two groups using propensity-matched analysis and the risk factors for anastomotic leakage were evaluated using logistic regression analyses.

**Results:**

Of the 121 patients, 33 were included in each group after propensity score matching. The clinicopathological characteristics of patients did not differ between the two groups after propensity score matching. In terms of perioperative outcomes, a significantly higher proportion of patients who underwent surgery using the laparoscopic approach (*P* < 0.001) and narrow gastric tube (*P* = 0.003), as well as those who had a lower volume of blood loss (*P* = 0.009) in the ICG circular group were observed after matching. Moreover, the ICG circular group had a significantly lower incidence of anastomotic leakage (39% vs. 9%, *P* = 0.004) and anastomotic stenosis (46% vs. 21%, *P* = 0.037) and a shorter postoperative hospital stay (30 vs. 20 days, *P* < 0.001) than the classical group. According to the multivariate analysis, the anastomotic method was an independent risk factor for anastomotic leakage after thoracoscopic esophagectomy (*P* = 0.013).

**Conclusions:**

Circular stapling anastomosis with ICG fluorescence imaging is effective in reducing complications such as anastomotic leakage and stenosis.

## Background

Esophageal cancer is the ninth most commonly diagnosed cancer worldwide and the sixth most common cause of cancer-related mortality [1]. Esophagectomy is the mainstay of treatment for resectable esophageal cancer. Thoracoscopic esophagectomy was first reported by Cuschieri et al. in 1992 [2] and has been used worldwide to a large extent as a curative surgery for esophageal cancer. It was reported to reduce the incidence of respiratory complications compared with open esophagectomy in a randomized controlled trial [3]. However, complications, including anastomotic leakage and stenosis, are a major cause of concern. At our institution, thoracoscopic esophagectomy was initially performed in November 2009 and this procedure has been standardized; however, the anastomotic method was not standardized until June 2018. In fact, various anastomotic methods, such as hand-sewn, triangulating [4], circular stapling, and Collard anastomosis [5], have been performed, but the complication rate of anastomosis has not reduced.

A systematic review reported that indocyanine green (ICG) fluorescence imaging could be an important adjunct tool for reducing anastomotic leakage following esophagectomy [6]. ICG is a water-soluble near-infrared phosphor that has immediate and long-term safety [7, 8]. ICG fluorescence imaging is a simple evaluation method. In a recent robot-assisted surgery, high-resolution near-infrared images were obtained by employing the Firefly system with the da Vinci Xi surgical robot (Intuitive Surgical Inc., Sunnyvale, California). Therefore, in order to assess the blood flow in the gastric tube during esophagectomy reconstruction, the use of ICG fluorescence imaging was standardized at our institution in July 2018. In addition, the anastomotic method was standardized to circular stapling anastomosis because it is simple and applicable to nearly all cases, including those with a short remnant esophagus.

This study aimed to evaluate the efficacy of circular stapling anastomosis with ICG fluorescence imaging for cervical esophagogastric anastomosis after thoracoscopic esophagectomy by comparing the short-term outcomes before and after the anastomotic method was standardized via a propensity-matched analysis.

## Methods

### Patients

**Fig. 1:**
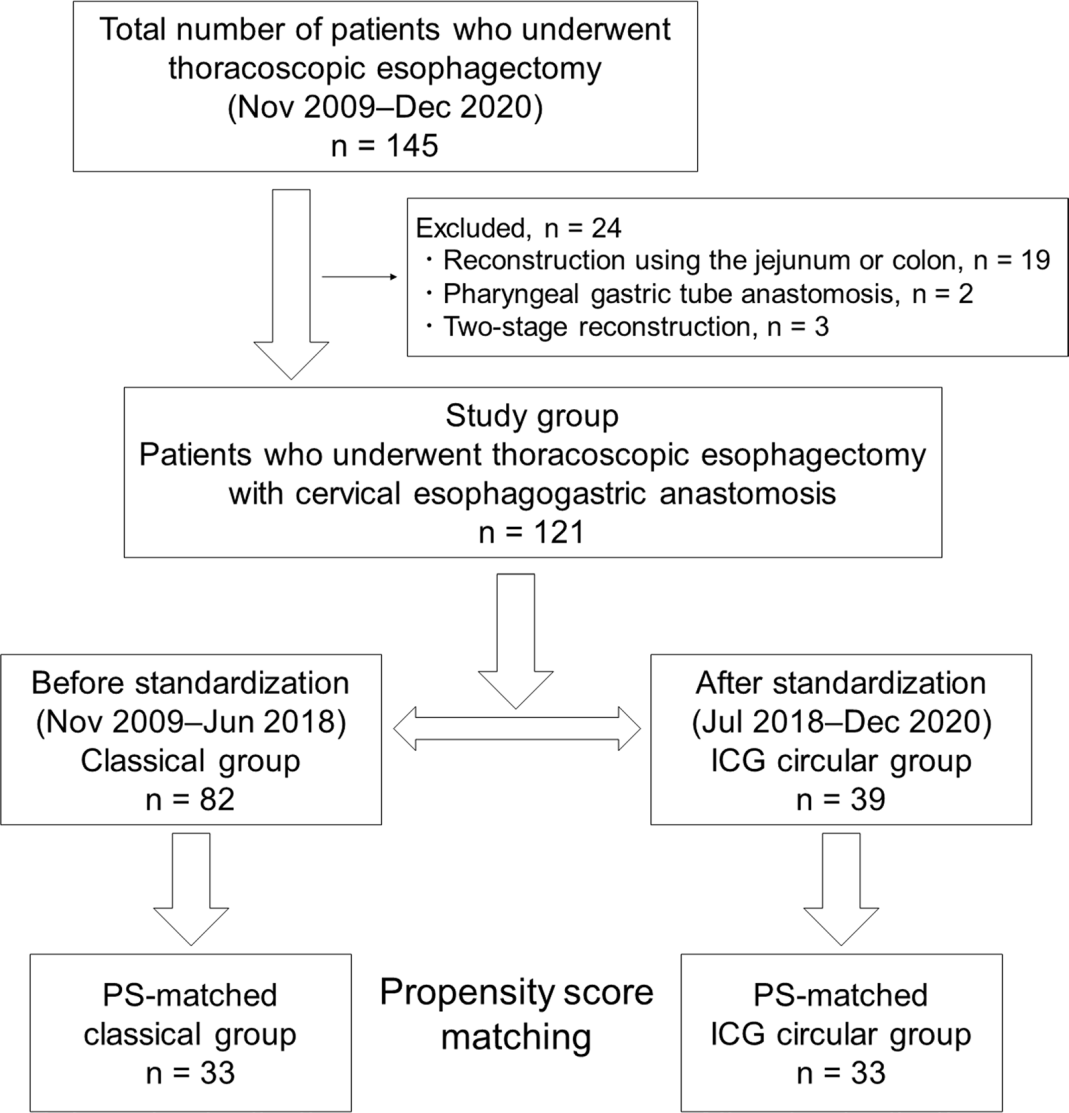
Patient selection for the evaluation of cervical esophagogastric anastomosis after thoracoscopic esophagectomy

Altogether, 145 patients with esophageal cancer who underwent thoracoscopic esophagectomy with radical lymph node dissection between November 2009 and December 2020 at Tottori University Hospital were included in this study. Among them, 19, 2, and 3 patients who underwent reconstruction using the jejunum or colon, pharyngeal gastric tube anastomosis caused by the simultaneous duplication of hypopharyngeal cancer, and two-stage reconstruction, respectively, were excluded. Finally, 121 patients were enrolled in this study (Fig. 1). Patients who underwent surgery until June 2018, i.e., before the standardization of the anastomotic method, were included in the classical group, and those who underwent surgery from July 2018, i.e., after the standardization of the anastomotic method, were included in the ICG circular group. The clinicopathological findings were determined as per the Japanese Classification of Esophageal Cancer (11th edition) [9, 10]. This study was approved by the institutional review board of Tottori University School of Medicine (20A234), and the requirement for informed consent was waived.

### Surgical procedure

All patients underwent thoracoscopic subtotal esophagectomy with mediastinal lymph node dissection in the prone position under right pneumothorax, and robot-assisted esophagectomy has been used since February 2020. After completion of the thoracic procedure, patients were repositioned in the supine position, and cervical and abdominal procedures were simultaneously initiated. Cervical lymph node dissection was not performed in patients with lower thoracic or abdominal esophageal cancer without cervical or upper mediastinal lymph node metastasis. Abdominal procedures, such as laparotomy, hand-assisted laparoscopic surgery, and complete laparoscopy for abdominal lymph node dissection, were performed. Patients who performed complete laparoscopy underwent laparotomy by making an incision of 8 cm in the upper abdomen after completing abdominal lymph node dissection, and the gastric tube was created under direct visualization in all cases. We created the gastric tube with a wide or narrow shape; the wide gastric tube is a method of resecting the stomach just below the esophagogastric junction, and the narrow gastric tube is a method of resecting the lesser curvature of the stomach so that the diameter of the gastric tube is approximately 3.5 cm. It was then pulled up to the neck through the retrosternal or posterior mediastinal route, and esophagogastric anastomosis was performed on the left side of the neck. In the classical group, before the anastomosis method was standardized, additional Kocher mobilization was performed as required for use until the area with good visual blood flow as the reconstructed gastric tube.

### ICG circular anastomosis method

**Fig. 2:**
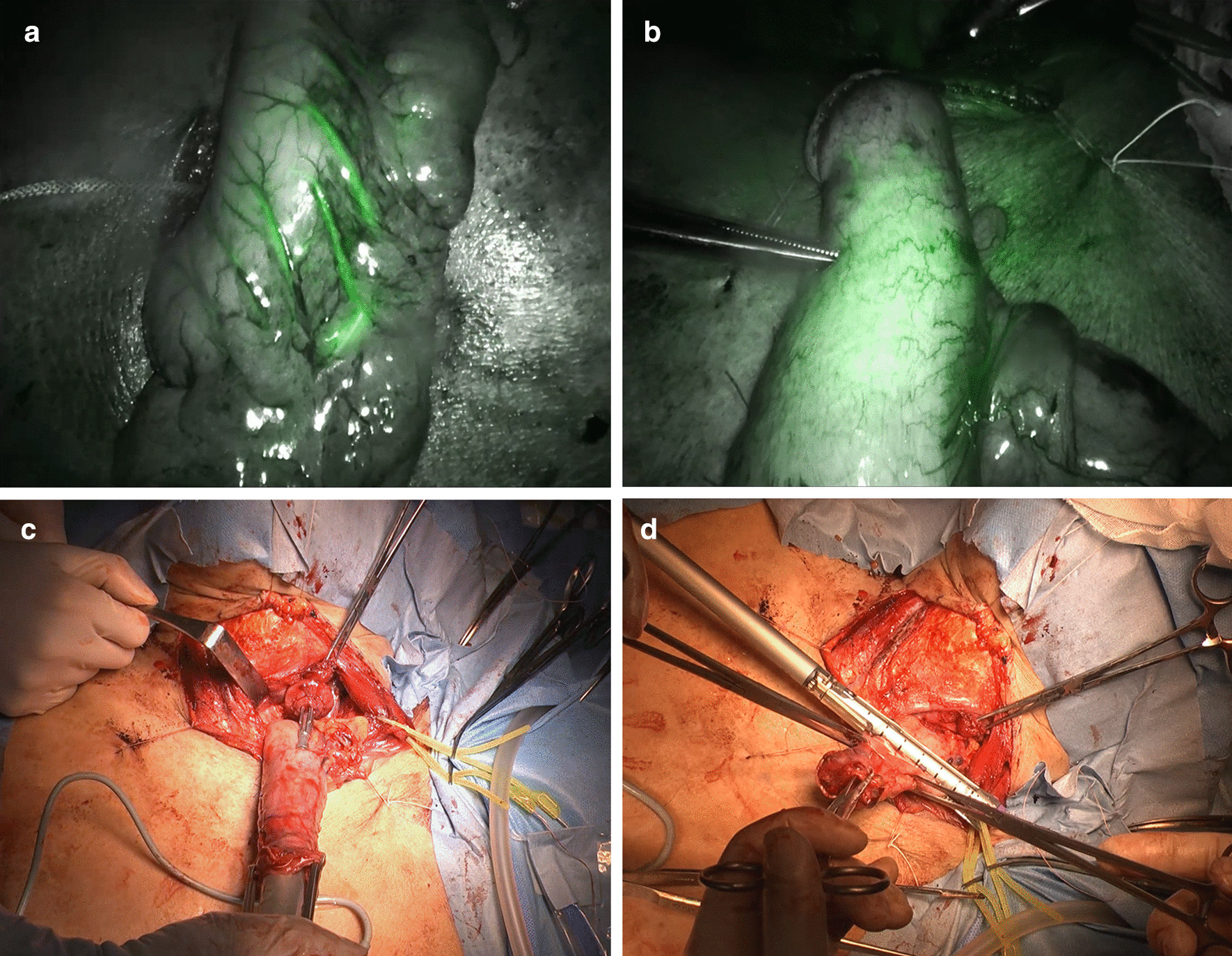
Procedures in indocyanine green (ICG) circular anastomosis. **a** The right gastroepiploic artery was contrasted with ICG; **b** The site where the wall of the gastric tube had a uniform contrast with ICG; **c** End-to-side esophagogastric anastomosis was performed on the posterior wall of the gastric tube using a 25-mm DST Series EEA circular stapler; **d** The stump of the gastric tube was sectioned and closed using the Signia stapling system with a 60 mm purple cartridge

The ICG circular anastomosis approach was used as follows: After the gastric tube was created, ICG at a dose of 10 mg/body was administered intravenously, and ICG fluorescence imaging of the blood flow in the gastric tube was assessed using the PhotoDynamic Eye (Hamamatsu Photonics, Hamamatsu, Japan) or the Firefly system, which was integrated with the da Vinci Xi surgical robot (Intuitive Surgical Inc., Sunnyvale, California). The reconstructed gastric tube was used until the site where the wall of the gastric tube had a uniform contrast within 20 s after contrasting the right gastroepiploic artery with ICG, as reported by Noma et al. [11] (Fig. 2a, b). After the gastric tube was pulled up to the neck, end-to-side esophagogastric anastomosis was performed on the posterior wall of the gastric tube using a 25-mm DST Series EEA circular stapler (Medtronic, Minneapolis, Minnesota) (Fig. 2c). The stump of the gastric tube was sectioned and closed using the Signia stapling system with a 60-mm purple cartridge (Medtronic, Minneapolis, Minnesota) (Fig. 2d). The staple line was lastly buried.

### Definition of perioperative complications

Anastomotic leakage was defined as saliva leakage from the cervical wound, contrast leakage outside the gastrointestinal tract on gastrointestinal series, and abnormal air or fluid accumulation around the site of anastomosis on computed tomography (CT) scan. A routine gastrointestinal series was performed on postoperative day 7; oral intake was initiated on postoperative day 8 for patients who experienced no problems in the postoperative course. This protocol maintained as is during the study period. Anastomotic stenosis was defined as cases in which an endoscope of 9.0 mm diameter could not pass through the anastomosis and balloon dilation was required during endoscopy for postoperative dysphagia. Pneumonia was defined as the appearance of consolidation on chest radiography or CT scan and the detection of bacteria on sputum culture. Recurrent nerve paralysis was assessed by an otolaryngologist on postoperative day 6 or 7 via laryngoscopy. The follow-up period for postoperative complications was 1 year postoperatively for anastomotic stenosis and until postoperative day 30 for other complications.

### Statistical analysis

Continuous data were presented as mean ± standard deviation or median with quartiles, as indicated. The Mann–Whitney *U*-test and the χ^2^ test were used to evaluate differences in continuous and categorical variables, respectively. A propensity-matched analysis was conducted using the logistic regression model and covariates such as age, sex, histological type, tumor location, clinical stage, and presence or absence of neoadjuvant chemotherapy. Univariate and multivariate logistic regression analyses were used to identify the risk factors for anastomotic leakage. Variables that were considered statistically significant in the univariate analysis were used for the multivariate analysis. *P* values of < 0.05 indicated statistically significant differences, and the Statistical Package for the Social Sciences software version 25 (IBM SPSS Inc., Chicago, Illinois) was used for statistical analyses.

## Results

### Characteristics of patients

Of 121 patients, 82 were included in the classical group and 39 in the ICG circular group before matching. Next, 33 patients were included in each group after matching (Fig. 1). Table 1 shows the clinicopathological characteristics of patients before and after matching. Before matching, significant differences were noted in terms of the American Society of Anesthesiologists physical status (ASA-PS) score (*P* = 0.021) and histological type (*P* = 0.009). However, after matching, the background characteristics did not significantly differ between the two groups.

**Table 1:** Characteristics of patients

	Before matching			After matching		
	Classical group	ICG circular group	*P* value	Classical group	ICG circular group	*P* value
	(n = 82)	(n = 39)		(n = 33)	(n = 33)	
Age (years)			0.892			0.872
Median (quartiles)	66 (61–72)	66 (61–71)		65 (61–73)	67 (63–73)	
Sex			0.914			0.282
Male	70 (85%)	33 (85%)		30 (90%)	27 (82%)	
Female	12 (15%)	6 (15%)		3 (9%)	6 (18%)	
Body mass index (kg/m^2^)	21.2 ± 3.1	22.2 ± 3.3	0.083	21.8 ± 3.1	21.9 ± 3.3	0.677
Serum albumin level (g/dL)	4.2 ± 0.4	4.1 ± 0.4	0.494	4.1 ± 0.4	4.1 ± 0.4	0.985
Brinkman index			0.733			0.278
Median (quartiles)	800 (445–1000)	860 (435–1000)		820 (600–1140)	840 (405–1000)	
ECOG performance status			0.586			0.601
0	68 (83%)	34 (87%)		27 (82%)	28 (85%)	
1	12 (15%)	5 (13%)		5 (15%)	5 (15%)	
2	2 (2%)	0 (0%)		1 (3%)	0 (0%)	
Comorbidity						
Diabetes	15 (18%)	4 (10%)	0.256	10 (30%)	4 (12%)	0.071
Cardiovascular disease	10 (12%)	6 (15%)	0.628	2 (6%)	5 (15%)	0.230
Obstructive ventilation failure	27 (33%)	13 (33%)	0.965	10 (30%)	11 (33%)	0.792
ASA-PS score			0.021			0.115
1	11 (13%)	1 (3%)		3 (9%)	1 (3%)	
2	63 (77%)	28 (72%)		27 (82%)	23 (70%)	
3	8 (10%)	10 (26%)		3 (9%)	9 (27%)	
Histological type			0.009			1.000
Squamous cell carcinoma	78 (95%)	30 (77%)		30 (91%)	30 (91%)	
Adenocarcinoma	2 (2%)	6 (15%)		2 (6%)	2 (6%)	
Others	2 (2%)	3 (8%)		1 (3%)	1 (3%)	
Tumor location			0.229			0.642
Upper thoracic	11 (13%)	6 (15%)		5 (15%)	6 (18%)	
Middle thoracic	43 (52%)	16 (41%)		15 (46%)	16 (49%)	
Lower thoracic	24 (29%)	11 (28%)		11 (33%)	7 (21%)	
Abdominal	4 (5%)	6 (15%)		2 (6%)	4 (12%)	
cT			0.405			0.667
1	36 (44%)	16 (41%)		16 (49%)	14 (42%)	
2	14 (17%)	11 (28%)		5 (15%)	9 (27%)	
3	31 (38%)	11 (28%)		11 (33%)	9 (27%)	
4a	1 (1%)	1 (3%)		1 (3%)	1 (3%)	
cN			0.224			0.420
0	44 (54%)	28 (72%)		19 (58%)	24 (73%)	
1	17 (21%)	5 (13%)		7 (21%)	4 (12%)	
2	20 (24%)	5 (13%)		7 (21%)	5 (15%)	
3	1 (1%)	1 (3%)		0 (0%)	0 (0%)	
cStage			0.296			0.393
1	32 (39%)	14 (36%)		16 (49%)	13 (39%)	
2	19 (23%)	14 (36%)		5 (15%)	10 (30%)	
3	31 (38%)	11 (28%)		12 (36%)	10 (30%)	
Neoadjuvant chemotherapy			0.974			1.000
Absent	36 (44%)	17 (44%)		16 (49%)	16 (49%)	
Present	46 (56%)	22 (56%)		17 (52%)	17 (52%)	

*ECOG* Eastern Cooperative Oncology Group, *ASA-PS* American Society of Anesthesiologists physical status

### Changes in anastomotic methods and perioperative outcomes

**Fig. 3:**
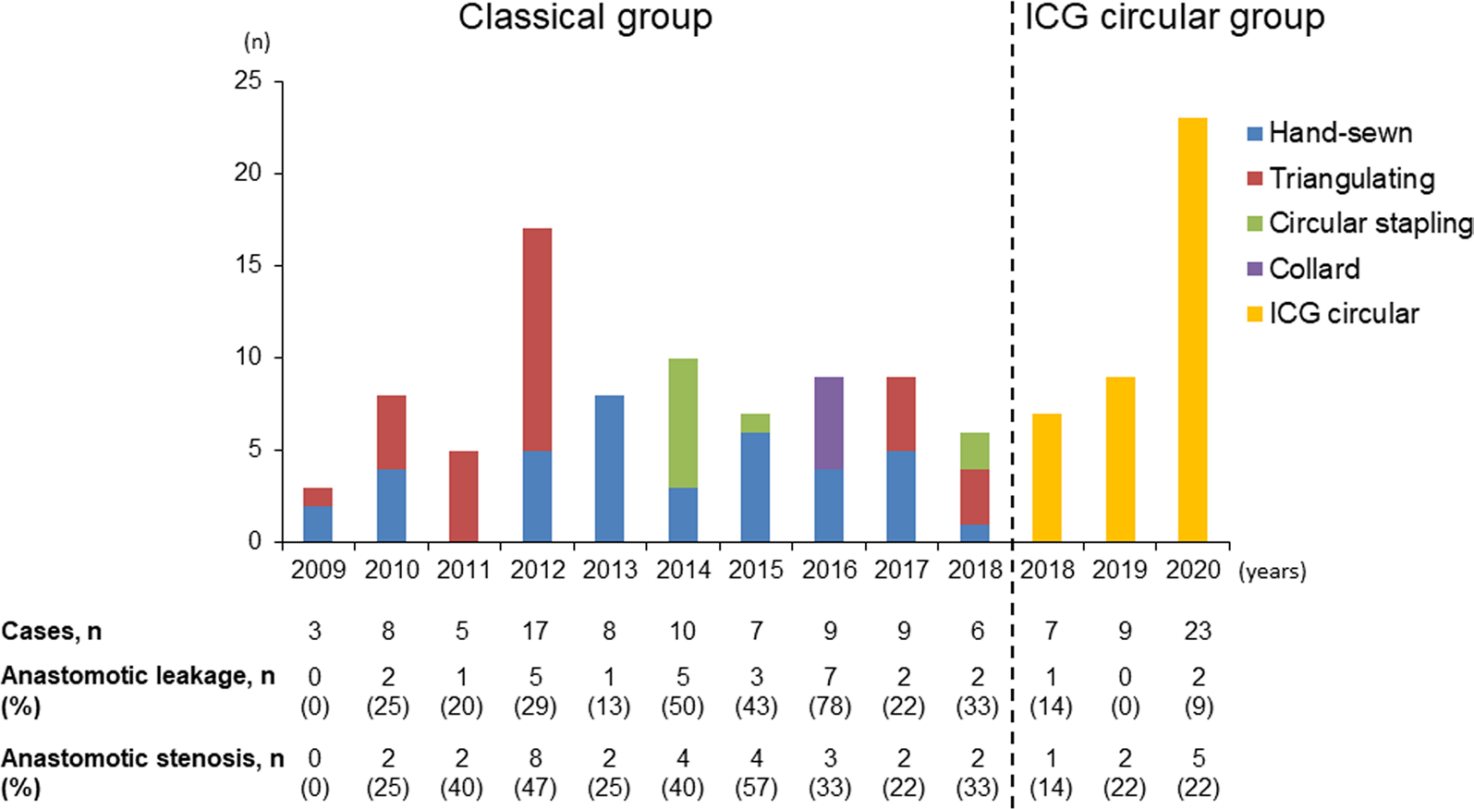
Changes in anastomotic methods for cervical esophagogastric anastomosis after thoracoscopic esophagectomy. Changes in the classical and ICG circular groups, and the annual incidence rates of anastomotic leakage and stenosis in anastomotic methods for cervical esophagogastric anastomosis after thoracoscopic esophagectomy are shown

Figure 3 presents changes in anastomotic methods for cervical esophagogastric anastomosis after thoracoscopic esophagectomy, and Table 2 depicts the perioperative outcomes in both groups. As shown in Table 2, prior to matching, a significantly higher proportion of patients underwent surgery using the laparoscopic approach (*P* < 0.001) and narrow gastric tube (*P* = 0.001) and those who had a lower volume of blood loss (*P* = 0.038) in the ICG circular group. After matching, the same factors indicated a significant difference. In terms of postoperative outcomes, the ICG circular group had a significantly lower proportion of patients who were observed to have anastomotic leakage (34% vs. 8%, *P* = 0.002) and a shorter postoperative hospital stay (29 vs. 20 days, *P* < 0.001) before matching. After matching, a significantly lower proportion of patients were noted to have anastomotic leakage (39% vs. 9%, *P* = 0.004) and stenosis (46% vs. 21%, *P* = 0.037) and a shorter postoperative hospital stay (30 vs. 20 days, *P* < 0.001) in the ICG circular group.

**Table 2:** Perioperative outcomes of patients with esophageal cancer after thoracoscopic esophagectomy

	Before matching			After matching		
	Classical group	ICG circular group	*P* value	Classical group	ICG circular group	*P* value
	(n = 82)	(n = 39)		(n = 33)	(n = 33)	
Abdominal approach			< 0.001			< 0.001
Open	34 (42%)	0 (0%)		15 (46%)	0 (0%)	
Laparoscopic	48 (59%)	39 (100%)		18 (55%)	33 (100%)	
Lymph node dissection			0.180			1.000
Two-field	18 (22%)	13 (33%)		10 (30%)	10 (30%)	
Three-field	64 (78%)	26 (67%)		23 (70%)	23 (70%)	
Route of reconstruction			0.070			0.131
Retrosternal	68 (83%)	37 (95%)		27 (82%)	31 (94%)	
Posterior mediastinal	14 (17%)	2 (5%)		6 (18%)	2 (6%)	
Shape of the gastric tube			0.001			0.003
Wide	21 (26%)	0 (0%)		8 (24%)	0 (0%)	
Narrow	61 (74%)	39 (100%)		25 (76%)	33 (100%)	
Total operative time (min)	634 ± 89	617 ± 53	0.573	638 ± 100	616 ± 51	0.753
Volume of blood loss (mL)	186 ± 219	103 ± 97	0.038	251 ± 298	93 ± 89	0.009
Postoperative complications						
Anastomotic leakage	28 (34%)	3 (8%)	0.002	13 (39%)	3 (9%)	0.004
Anastomotic stenosis	29 (35%)	8 (21%)	0.097	15 (46%)	7 (21%)	0.037
Pneumonia	18 (22%)	11 (28%)	0.451	8 (24%)	9 (27%)	0.778
Recurrent nerve paralysis	14 (17%)	2 (5%)	0.070	7 (21%)	2 (6%)	0.073
Postoperative hospital stay	29 (22–44)	20 (16–28)	< 0.001	30 (25–44)	20 (17–28)	< 0.001

### Risk factor analyses of anastomotic leakage

Finally, the risk factors for anastomotic leakage were evaluated via propensity score matching in 66 patients. The univariate analysis indicated that the Brinkman index (*P* = 0.048) and anastomotic method (*P* = 0.008) were significantly associated with anastomotic leakage (Table 4). According to the multivariate analysis, the anastomotic method was an independent risk factor for anastomotic leakage after thoracoscopic esophagectomy (odds ratio: 5.983, 95% confidence interval (CI): 1.469–24.359, *P* = 0.013) (Table 3).

**Table 3:** Univariate logistic regression analyses of anastomotic leakage

	Anastomotic leakage				
	Absent (n = 50)	Present (n = 16)	OR	95% CI	*P* value
Age (years)					
<65	20 (40%)	8 (50%)	1.500	0.484–4.651	0.483
≥65	30 (60%)	8 (50%)	1		
Sex					
Male	42 (84%)	15 (94%)	2.857	0.329–24.795	0.341
Female	8 (16%)	1 (6%)	1		
Body mass index (kg/m^2^)					
<22	24 (48%)	10 (63%)	1.806	0.569–5.726	0.316
≥ 22	26 (52%)	6 (38%)	1		
Serum albumin level (g/dL)					
<4	20 (40%)	5 (31%)	0.682	0.206–2.261	0.531
≥ 4	30 (60%)	11 (69%)	1		
Brinkman index					
<800	24 (48%)	3 (19%)	0.250	0.063–0.986	0.048
≥ 800	26 (52%)	13 (81%)	1		
Performance status					
0	42 (84%)	13 (81%)	0.825	0.191–3.574	0.797
1, 2	8 (16%)	3 (19%)	1		
Diabetes					
Absent	39 (78%)	13 (81%)	1.222	0.295–5.069	0.782
Present	11 (22%)	3 (19%)	1		
Cardiovascular disease					
Absent	46 (92%)	13 (81%)	0.377	0.075–1.901	0.237
Present	4 (8%)	3 (19%)			
Obstructive ventilation failure					
Absent	36 (72%)	9 (56%)	0.500	0.156–1.603	0.243
Present	14 (28%)	7 (44%)	1		
ASA-PS score					
1	3 (6%)	1 (6%)	1.044	0.101–10.806	0.971
2, 3	47 (94%)	15 (94%)	1		
Histological type					
Squamous cell carcinoma	45 (90%)	15 (94%)	1.667	0.180–15.425	0.653
Others	5 (10%)	1 (6%)	1		
Tumor location					
Ut, Mt	31 (62%)	11 (69%)	1.348	0.406–4.484	0.626
Lt, Ae	19 (38%)	5 (31%)	1		
cT					
1	21 (42%)	9 (56%)	1.776	0.570–5.531	0.322
2, 3, 4a	29 (58%)	7 (44%)	1		
cN					
Absent	33 (66%)	10 (63%)	0.859	0.267–2.764	0.798
Present	17 (34%)	6 (38%)	1		
cStage					
1	20 (40%)	9 (56%)	1.929	0.618–6.020	0.258
2, 3	30 (60%)	7 (44%)	1		
Neoadjuvant chemotherapy					
Absent	22 (44%)	10 (63%)	2.121	0.668–6.739	0.202
Present	28 (56%)	6 (38%)	1		
Abdominal approach					
Open	11 (22%)	4 (25%)	1.182	0.317–4.400	0.803
Laparoscopic	39 (78%)	12 (75%)	1		
Lymph node dissection					
Two-field	15 (30%)	5 (31%)	1.061	0.314–3.585	0.925
Three-field	35 (70%)	11 (69%)	1		
Route of reconstruction					
Retrosternal	43 (86%)	15 (94%)	2.442	0.277–21.519	0.421
Posterior mediastinal	7 (14%)	1 (6%)	1		
Shape of gastric tube					
Wide	5 (10%)	3 (19%)	2.077	0.437–9.871	0.358
Narrow	45 (90%)	13 (81%)	1		
Total operative time (min)					
<600	20 (40%)	6 (38%)	0.900	0.282–2.870	0.859
≥ 600	30 (60%)	10 (63%)	1		
Blood loss (mL)					
<100	26 (52%)	7 (44%)	0.718	0.231–2.229	0.566
≥ 100	24 (48%)	9 (56%)	1		
Anastomotic method					
ICG circular group	30 (60%)	3 (19%)	0.154	0.039–0.610	0.008
Classical group	20 (40%)	13 (81%)	1		

*OR* odds ratio, *CI* confidence interval, *ASA-PS* American Society of Anesthesiologists physical status

**Table 4:** Multivariate logistic regression analyses of anastomotic leakage

	OR	95% CI	*P* value
Brinkman index (≥ 800)	3.538	0.842–14.860	0.084
Anastomotic method (classical group)	5.983	1.469–24.359	0.013

*OR* odds ratio, *CI* confidence interval

## Discussion

This study aimed to evaluate the efficacy of circular stapling anastomosis with ICG fluorescence imaging for cervical esophagogastric anastomosis after thoracoscopic esophagectomy by comparing the short-term outcomes before and after the standardization of the anastomotic method via a propensity-matched analysis. The ICG circular group had a significantly lower rate of complications, including anastomotic leakage and stenosis, and a shorter postoperative hospital stay. Furthermore, anastomotic method was an independent risk factor for anastomotic leakage after thoracoscopic esophagectomy.

The incidence of anastomotic leakage was significantly lower in the ICG circular group than in the classical group, and anastomotic method was found to be an independent risk factor for postoperative anastomotic leakage. According to a systematic review on ICG fluorescence imaging after esophageal cancer surgery, the overall anastomotic leakage rate in patients who underwent surgery with ICG fluorescence imaging was lower than that in controls (13.5% [118/873] vs. 18.5% [86/466]) [6]. Furthermore, when anastomosis was performed at the site where good ICG perfusion was observed, the incidence of anastomotic leakage was 9.0% (67/746). In this study, anastomosis was performed at the site where good ICG perfusion was observed in all patients in the ICG circular group. Thus, the outcomes of anastomotic leakage were comparable. Honda et al. performed a systematic review comparing outcomes of anastomosis after esophagectomy between hand-sewn and mechanical anastomosis using a circular stapler [12]. Results showed that the anastomotic leakage rate with circular stapling anastomosis (6.1%, 41/668) was similar to that with hand-sewn anastomosis (6.1%, 39/640) (risk ratio [RR]: 1.02, 95% CI: 0.66–1.59, *P* = 0.43); compared with our results, particularly in classical group, the rate of anastomotic leakage was extremely low. The most important reason for the high anastomotic leakage rate in the classical group was that an excessive number of anastomosis methods were performed. However, in the ICG circular group, anastomosis was performed using a completely uniform technique in all cases, which may have had a substantial impact on our results. Moreover, the review by Honda et al. included studies in which intrathoracic anastomosis was performed. Thus, the outcomes of this review were not completely comparable to those of cervical esophagogastric anastomosis, and our results were acceptable. In addition, Honda et al. showed that the approach using mechanical circular stapling anastomosis considerably reduced the operative time by 15.3 min compared with that required by hand-sewn anastomosis. In our study, the total operative time tended to be shorter in the ICG circular group than in the classical group; however, it was not significant. Therefore, circular stapling anastomosis with ICG fluorescence imaging is a simple and safe method that can reduce anastomotic leakage after thoracoscopic esophagectomy.

This study showed that the incidence of anastomotic stenosis was substantially lower in the ICG circular group than in the classical group after propensity score matching. However, the anastomotic stenosis rate in the classical group after matching was extremely high at 46%, which may have had a significant impact. Honda et al., who conducted a subgroup and meta-regression analysis, showed that the stenosis rate of circular stapling anastomosis was significantly higher than that of hand-sewn anastomosis (16.9% [106/626] and 9.9% [62/629]) (RR: 1.67, 95% CI: 1.16–2.42; *P* = 0.006); they showed no significant differences in the anastomotic site, diameter of the circular stapler, layer, and configuration [12]. A randomized controlled trial by Hayata et al. comparing cervical esophagogastric circular stapling and triangulating stapling anastomoses after esophagectomy showed no significant difference in terms of anastomotic stenosis rate between the circular stapling group (17%, 8/47) and the triangulating stapling group (19%, 9/51) (*P* = 0.935) [13]. In this study, the anastomotic stenosis rate in the ICG circular group was 21%, which was similar to that noted in previous reports but was not satisfactory. Therefore, although the incidence rate of anastomotic stenosis is improving because of the standardization of anastomotic methods, it should still be further reduced.

This study had several limitations. First, this was a retrospective study with a small sample size. All relevant clinicopathological and technical factors, with the exception of the anastomotic method, should have been included in the matching factors; however, this was not practical because of the small sample size. As a result, reconstruction-related perioperative outcomes, such as abdominal approach and shape of gastric tube were not consistent between the two groups. Therefore, the outcomes of ICG circular anastomosis after standardization must be prospectively evaluated. Second, various anastomotic methods were performed in the classical group patients. Therefore, a propensity-matched analysis was performed to eliminate differences in patient characteristics and to prevent bias as much as possible.

## Conclusions

Circular stapling anastomosis with ICG fluorescence imaging was found to be effective in reducing anastomotic complications for cervical esophagogastric anastomosis after thoracoscopic esophagectomy. It was particularly crucial for the anastomotic method to be standardized in this study. However, the incidence rate of anastomotic stenosis can still be improved, and this is a problem that should be addressed in the future.

## Data Availability

The datasets generated and analyzed during the current study are not publicly available due to difficulty in obtaining approval from all patients for the release of detailed personal data, but they are available from the corresponding author on reasonable request.

## Abbreviations

ICG: Indocyanine green
ASA-PS: The American Society of Anesthesiologists physical status
CI: Confidence interval
RR: Risk ratio
